# Cardio-respiratory interactions in acute asthma

**DOI:** 10.3389/fphys.2023.1232345

**Published:** 2023-09-15

**Authors:** Morgan Caplan, Olfa Hamzaoui

**Affiliations:** ^1^ Service de Médecine Intensive Réanimation, Hôpital Robert Debré, Université de Reims, Reims, France; ^2^ Unité HERVI, Hémostase et Remodelage Vasculaire Post-Ischémie, Reims, France

**Keywords:** asthma, heart-lung interaction, spontaneous ventilation, pleural pressure, hyperinflation dynamics

## Abstract

Asthma encompasses of respiratory symptoms that occur intermittently and with varying intensity accompanied by reversible expiratory airflow limitation. In acute exacerbations, it can be life-threatening due to its impact on ventilatory mechanics. Moreover, asthma has significant effects on the cardiovascular system, primarily through heart-lung interaction-based mechanisms. Dynamic hyperinflation and increased work of breathing caused by a sharp drop in pleural pressure, can affect cardiac function and cardiac output through different mechanisms. These mechanisms include an abrupt increase in venous return, elevated right ventricular afterload and interdependence between the left and right ventricle. Additionally, Pulsus paradoxus, which reflects the maximum consequences of this heart lung interaction when intrathoracic pressure swings are exaggerated, may serve as a convenient bedside tool to assess the severity of acute asthma acute exacerbation and its response to therapy.

## Introduction

Asthma is a heterogenous disease that affects over a quarter of a billion people worldwide (Anagnostou et al., 2012; Royal College of Physicians, 2014; Levy, 2015). It is characterized by a range of respiratory symptoms that occur variably over time with varying intensity. These symptoms are associated with reversible expiratory airflow limitation which can be confirmed by spirometry 15 min after administering a bronchodilator (Global initiative for Asthma, 2022). Despite advancements in care and the development of new therapies, it remains a public health problem. Its prevalence is increasing in many developing countries and it remains a leading cause of mortality worldwide particularly among children and young individuals (GBD, 2015 Chronic Respiratory Disease Collaborators, 2017; Brusselle and Koppelman, 2022; Global initiative for Asthma, 2022).

From a pathophysiological aspect, asthma is a syndrome exhibiting clinical and physiological heterogeneity. In acute exacerbations, defined by the change from the patient’s previous condition, which includes the onset or progression of symptoms and a decline in usual respiratory function asthma, asthma can be life-threatening through its impact on ventilatory mechanics. The decline can be measured by assessing parameters such as peak expiratory flow or forced expiratory volume in the first second (FEV1). Airway inflammation and airway hyperresponsiveness remain the cornerstones of the disease pathophysiology. These factors lead to a decrease in airway caliber and limitation of expiratory flows through various mechanisms (Kudo et al., 2013; Bush, 2019; Kaminsky and Chapman, 2020).

Furthermore, asthma has systemic effects, although the underlying mechanisms are not fully elucidated. Cardiac and vascular consequences are the most studied. The association between asthma and cardiovascular risk particularly in women has been well described (Iribarren et al., 2012; Strand et al., 2018). However, there is limited knowledge regarding the potential relationship between asthma and heart function. Previous echocardiographic studies have reported alterations in cardiac function in children and young adults even in the early stages of the disease (Shedeed, 2010; De-Paula et al., 2018; Di Raimondo et al., 2022; Karakaya et al., 2022). Using Tissue Doppler imaging, (Shedeed, 2010) highlighted the existence of right ventricular (RV) diastolic dysfunction which correlated with the severity of asthma in asthmatic children. These findings appear to be consistent with recent studies in adult population. In line with these observations, previous studies have reported various cardiac abnormalities in asthmatic patients such as an increase in indexed LV mass (Sun et al., 2017) and a significantly reduced left and right ventricular strain values in severe and mild-to-moderate asthma (Tulet et al., 2019). More recently, the BADA study-ECO has provided further evidence supporting the existence of significant subclinical cardiac involvement in asthma (Di Raimondo et al., 2022).

In this review, we will explore the consequences of airway narrowing in most severe form of acute asthma [Life-threatening asthma also referred to as near-fatal asthma, status asthmaticus or critical asthma syndrome (Kenyon et al., 2015)] and describe the cardio-pulmonary interactions that occur in spontaneous ventilation, ranging from basic lung inflation to the appearance of pulsus paradoxus which can occur during the most severe crisis.

### Repercussions of asthma on ventilatory mechanics and work of breathing

Spontaneous breathing relies on the reduction of intra-thoracic pressures (ITP) which results in an increase in lung volumes. The multifactorial airway narrowing is directly responsible of the symptomatology of acute asthma. The reduction in airway caliber leads to an increase workload for breathing due to high flow-related resistance especially in inspiration. Increased airway resistance results in low inspiratory and expiratory flow rates (FEV1 and Peak Expiratory Flow) and premature closure of small airways, despite the positive transpulmonary pressure (i.e., alveolar pressure—pleural pressure (Ppl)) (Rodriguez-Roisin, 1997). In acute severe asthma, lung hyperinflation occurs because the respiratory system cannot reach static equilibrium volume at the end of expiration. This is attributed to several factors including increased airway expiratory resistance, high ventilatory demand, short expiratory time, and increased post-inspiratory activity of the inspiratory muscles, all of which are present to varying degrees in status asthmaticus patients. As a result, inspiration begins at a volume where the respiratory system exhibits a positive recoil pressure. This pressure is known as intrinsic positive-end expiratory pressure (PEEPI) or auto-PEEP when the end expiratory lung volume is exceeding functional residual capacity (Rodriguez-Roisin, 1997). This phenomenon known as dynamic hyperinflation, is directly proportional to minute ventilation (V E) and the degree of airflow obstruction. These changes in lung volumes help to keep constricted airways opened and increase transpulmonary pressure. Tidal breathing occurs near predicted total lung capacity to maintain flow rates which can be beneficial since it improves gas exchange (Whea et al., 1990). This phenomenon requires the generation of strongly negative ITP to maintain minute ventilation and maintains the lung at higher lung volume with large negative peak inspiratory and mean pressure during the breathing cycle (Stalcup and Mellins, 1997).

However, dynamic hyperinflation has significant adverse effects on lung mechanics. It increases the three components of respiratory system load, namely, resistance, elastance, and minute volume. First, the shift of the tidal breathing to a less compliant part of the pressure-volume curve leads to an increase of work of breathing. Secondly, it flattens the diaphragm and reduces muscle contraction due to a mechanically disadvantageous fiber length. Third, dynamic hyperinflation increases dead space, thereby requiring a higher minute volume to maintain adequate ventilation.

#### Repercussions of pleural pressure swings

Hyperinflation in acute asthma will influence hemodynamics and cardiac output through two distinct phenomena. The variation in transpulmonary pressure described above (pressure exerted on the lung wall representing the delta between alveolar pressure and pleural pressure (Ppl)) and Ppl swings generated at each inspiration to overcome airway flow limitation (Scharf et al., 1979).

1) On venous return

The force acting against an elastic structure is equal to the difference between the inside and the outside pressure of the elastic structure called transmural pressure. This concept applied to the heart, emphases the importance of Ppl variations during the respiratory cycle (Lai-Fook and Rodarte, 1991). According to Guyton et al. (1957), right atrial pressure serves as the opposing force to venous return and inspiration enhances the pressure gradient for systemic venous return (atrial pressure-mean systemic pressure). In acute asthma, the negative pleural inspiratory pressure generated to overcome resistance promotes venous return to the thorax by distending compliant right atrium leading to an increase in right heart filling. Simultaneously, diaphragmatic movement increases intra-abdominal pressure, facilitating blood flushing by an elevation of the gradient with the right atrium. If the right ventricle (RV) operates on the steep portion of Franck Starling’s curve (indicating preload dependency), the increased RV preload during inspiration amplifies stroke volume in accordance with the relationship between preload and stroke volume. Notably, the augmented pulmonary flow during inspiration occurs despite a minor increase in pulmonary vascular resistance (PVR) (BRECHER and HUBAY, 1955).

The lowering of Ppl cannot indefinitely increase venous return due to the development of a vascular waterfall phenomenon. Abdominal pressure and venous blood volume may influence inferior vena cava flow which carries most of the venous return (Takata et al., 1992). To illustrate, if the pressure in the right atrium becomes negative compared to atmospheric pressure, a decrease in Ppl will not enhance cardiac filling and inferior vena cava may collapse. In acute asthma, the right heart filling and the venous return will gradually be limited by the elastic properties of the RV within the pericardium. In case of RV dysfunction, an increase in venous return leads to elevated pressure without affecting RV volume or stroke volume since the RV operates on the flat part of the Frank Starling curve (preload independence).

2) On right ventricular afterload

The RV functions as a flow-generating pump rather than a pressure-generating pump as it ejects blood in a compliant vascular system. The negative ITP at inspiration may impede RV afterload. Indeed, the increased difference between negative ITP and intra-alveolar pressure (pressure surrounding the alveolar vessels) at the end of inspiration creates an obstacle to RV ejection.

Accordingly, the drop in Ppl may impede RV function as suggested in some physiological studies (Stalcup and Mellins, 1997). However, the pulmonary vasculature which is highly distensible, serves as a good surrogate to determine the RV afterload. In quiet respiration, variations in ITP do not affect PVR and alteration in RV output may be explained by alteration in cardiac performance (Koshino et al., 2010). These physiological mechanisms are nowadays evaluated with modern techniques. Analysis of RV strain during Muller maneuver, simulating a drop in Ppl, revealed an alteration in ventricular contractility (Koshino et al., 2010).

The decrease in Ppl during deep breathing affects the transmural pressure of the vessels and tends to distend inter-alveolar vessels. However, characterizing this relationship in acute asthma is challenging due to the heterogeneous phenomena of pulmonary inflation and auto-PEEP, which have been extensively described for their effects on pulmonary arterial pressures since the 1950s (BRECHER and HUBAY, 1955).

3) On left ventricular pre and afterload

During spontaneous breathing, the increase in right cardiac output during inspiration leads to a subsequent increase in left cardiac output after few beats (during expiration) due to a series connection.

During inspiration, the left heart located in the chest, experiences an increased transmural pressure and must pump blood towards the extra thoracic arteries. To compensate for the transmural pressure gradient between the ventricular wall and that of the extra-thoracic vessels, the left heart needs to generate higher pressures. The impact of deep breathing on left ventricular performance has been investigated physiologically using Muller’s maneuver (Buda et al., 1979). Negative ITP affects left ventricular afterload with a more or less marked effect depending on the timing of inspiration with respect to cardiac systole. Negative Ppl also plays a significant role in reducing left stroke volume (Scharf et al., 1981). The sometimes-discordant results point out the difficulties in the analyses of cardiopulmonary interactions according to the respiratory cycle and to the phases of systole and diastole (Brinker et al., 1980; Scharf et al., 1981). Peters et al. (1988a); Peters et al. (1988b) have well described the impact of inspiration in function of the cardiac cycle in animal model (Cheyne et al., 2016). They observed that a decrease in ITP during diastole tended to decrease stroke volume by reducing preload, while inspiration concomitant with systole tended to increase afterload (Peters et al., 1988a; Peters et al., 1988b). However, it is generally accepted that in a healthy heart and spontaneous ventilation, performance is more influenced by preload than afterload (Quinones et al).

In acute asthma, there is a significant swing towards negative values of Ppl (−25 cmH20) which is on average negative over the entire cycle. Buda et al. (1979) have reported that the maximum alteration of cardiac function via afterload occurs with ITP as low as −60 cmH_2_0 during Muller maneuver (Stalcup and Mellins, 1977; Buda et al., 1979). While the variation in ITP may not have a significant impact on the left ventricular afterload during asthma, this additional constraint coupled with increased RV filling and the elevation gradient across the pulmonary interstitium contributes to the cardiac consequences associated with asthma (Stalcup and Mellins, 1977; Timby et al., 1990).

#### Repercussions of transpulmonary pressures

1) On the right ventricle:

The relationship between lung inflation and cardiac function is complex. In cases of physiological or moderate inflation, the mediastinal constraint is limited, and the hemodynamic effects are generally attributed to changes in pulmonary resistance or to the decrease in Ppl as described in chronic obstructive pulmonary diseases (Cheyne et al., 2016). However, studies have demonstrated that lung inflation does have a mechanical effect on the diastolic filling of the RV (Brookhart and Boyd, 1947).

Alveolar pressures and lung volume influence hemodynamics through their effect on PVR. The effect of inflation depends on the distribution of the vessels, whether they are intra or extra-alveolar. Extra-alveolar vessels are located in lung parenchyma and consists of smooth muscles and elastic tissues. The resistance of those vessels decreases with lung distension due to radial traction on their walls.

**FIGURE 1:**
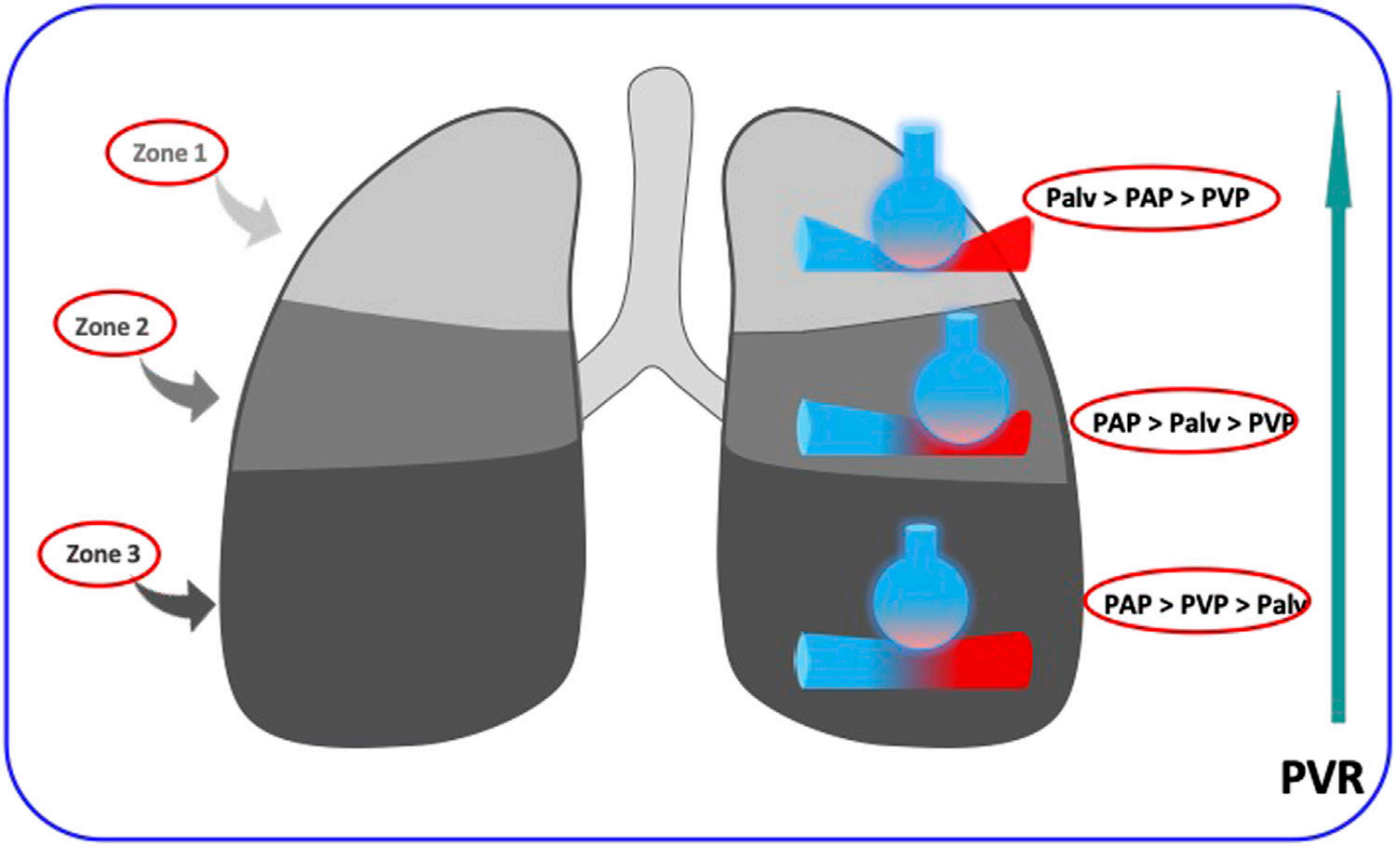
The increase in pulmonary vascular resistances (PVR) from Zone west 3 to zone West 1. in the upper zones (zones 1 of West), the alveolar pressure (Palv) is higher than the intravascular pressure (PAP and PVP) and occludes the intra-alveolar vessels, in the lower zones (zones 3 of West) the intravascular pressure is higher than the alveolar pressure and the vascular resistances are weak. The regimen is intermediate in the middle zones (zones 2 of West).

**FIGURE 2:**
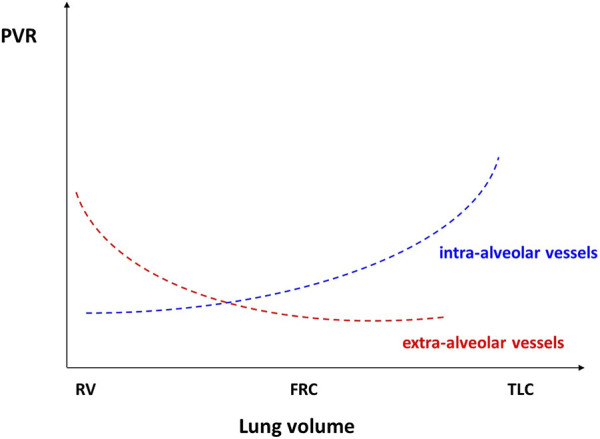
Reproduced with permission of the ^©^ ERS 2023: European Respiratory Journal December 2013, 42 (6) 1,696–1,705; DOI: 10.1183/09031936.00138912). Relationships between pulmonary vascular resistances and lung volume (see text for detailed explanations). FRC, functional residual capacity; TLC, total lung capacity; RV, residual volume.

The PVR of alveolar vessels (including capillaries) is primarily influenced by transmural pressure. In a ventilated and perfused dog lung model, West demonstrated the existence of different blood flow regions dependent on the levels of alveolar pressures and thus characterized the pulmonary model divided into 3 zones (Figure 1) according to their vascularization and their ventilation (West et al., 1964). During homogeneous and prolonged hyperinflation, the cyclical drops in Ppl promote the collapse of pulmonary vessels and hence the increase of the non-zone 3 units (West Zone I and 2). Inflation directly affects PVR, and the right cardiac afterload as observed in mechanical ventilation (Slobod et al., 2022). Consequently, the total pulmonary resistance describes a U-shaped pattern (Figure 2). Resistance is high at total lung capacity and decreases to the lowest value at functional residual capacity. It is established that PVR increases exponentially with lung volumes above functional residual capacity (Permutt et al., 1962).

2) On the left ventricle:

On the left side, pulmonary veins constitute the blood reservoir. Lung inflation squeezes blood out of the vessels in West zone III leading to an increase in left atrial filling pressures (Brower et al., 1985). This adds to the increase in left heart volume caused by the inspiratory rise in afterload as described previously. Cheyne et al. (2016) demonstrated that even a modest hyperinflation (defined by a reduction in inspiratory reserve volume) progressively decreased left ventricular stroke volume due to the progressive reduction of left ventricular end diastolic filling, likely because of increased PVR and interdependence between ventricles. In their study, the effect of hyperinflation appears to be cumulative with the effect of significant variations in Ppl on ventricular volumes.

##### Interdependence between ventricles

The right and the left ventricle are interdependent structures. They are nestled within the pericardium, share a common septum, and have common overlapping muscular fibers (Janicki and Weber, 1980). A forced inspiration results in an increased systemic venous return to the right side of the heart. This, in turn, causes an increase in RV end-diastolic volume. At the same time, if the filling pressure of the right ventricle rises, the septum shifts and reduces the compliance of the left ventricle during both systole and diastole (Brinker et al., 1980). At high levels of inflation and in hearts with greater volume, increased septal flattening suggests that left ventricular underfilling during hyperinflation may be primarily a result of ventricular interaction exacerbated by ventricular interdependence (Robotham et al., 1983). In a randomized controlled trial conducted by Urban et al. (2021), it was demonstrated that dynamic pulmonary hyperinflation has a notable impact on left heart diastolic function, expressed as the E/A ratio in echocardiography, and leads to an increase in ventricular filling pressures, as indicated by the E/e' ratio. They highlighted an inverse correlation between positive end expiratory pressure and E/A ratio. In addition, In the study by Jardin et al. (1982), the authors included 9 patients in status asthmaticus and examined hemodynamic changes using Pulmonary artery catheter (PAC) and echocardiography. The authors reported that left ventricular dimensions were smaller during inspiration and the left ventricular transmural filing pressure was significantly higher. Indeed, increased left ventricular filing pressure in the presence of decreased left ventricular dimension are in favor of a decreased compliance of the left ventricle due to the displacement of interventricular septum (Jardin et al., 1982).

The two ventricles of the heart constitute two pumps mounted in series and separated by the pulmonary vasculature. Thus, the influence of the respiratory cycle on the stroke volume of the right heart affects the left cardiac preload. During inspiration, negative swings in Ppl enhance venous return and RV filling. This increase is reflected on stroke volume after a few cardiac cycles. However, the variations in RV stroke volume are not strictly comparable to those of the LV given the compliance of the pulmonary vascularization (Magder and Guerard, 2012). The RV stroke volume reaches its maximum during inspiration while the increase in left ventricle stroke volume is highest in expiration, influenced by the pulmonary transit time. This serial interaction has been demonstrated in quiet spontaneous breathing subject as well as under mechanical ventilation and it can be useful to predict volume responsiveness (Ruskin et al., 1973; Michard et al., 2000). Nevertheless, clinical expression of this relationship needs to be further defined in acute asthma where tachycardia, increase in cardiac output, tachypnea and low tidal volumes are predominant (De Backer et al., 2005).

### Heart lung interactions during life-threatening asthma: pulsus paradoxus

**FIGURE 3:**
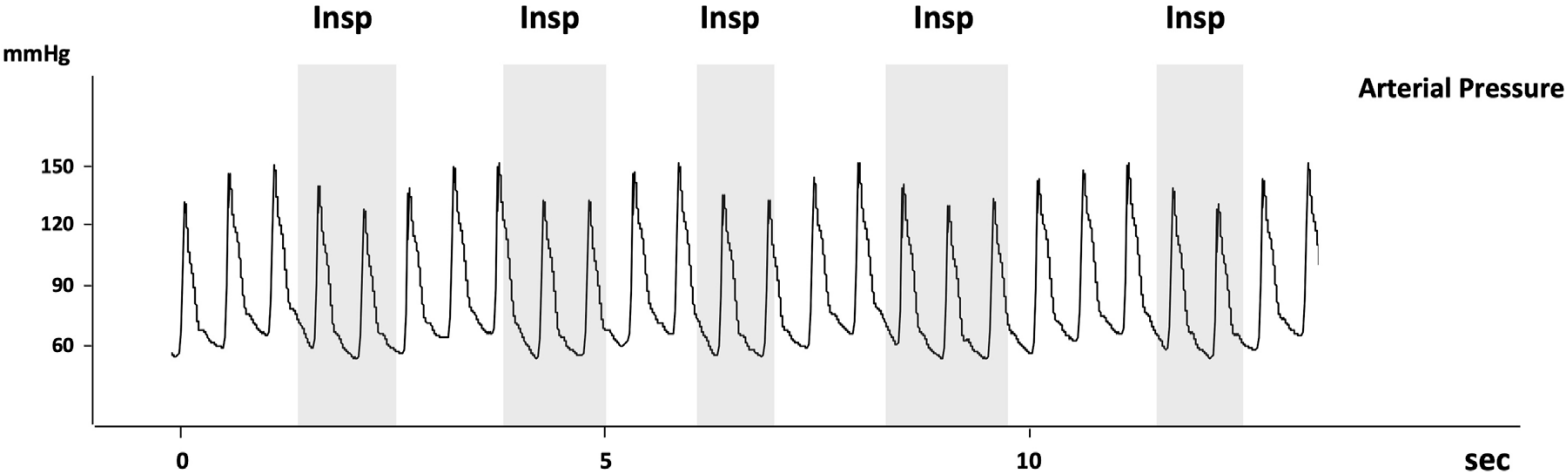
Reproduced with permission of the ^©^ ERS 2023: European Respiratory Journal December 2013, 42 (6) 1,696–1,705; DOI: 10.1183/09031936.00138912. Recording of arterial pressure measured using an arterial catheter in a patient with acute exacerbation of airway obstruction. Pulsus paradoxus is present as the decrease in systolic arterial pressure is >10 mmHg (about 17 mmHg). Note that the arterial pulse pressure also decreases at inspiration suggesting that LV stroke volume decreases at inspiration. The grey areas indicate the inspiratory phases (Insp).

During acute asthma, the inspiratory fall in Ppl and high-volume ventilation with dynamic hyperinflation may lead to the appearance of pulsus paradoxus indicating cardio-pulmonary interactions and reflecting the severity of airway obstruction (Knowles and Clark, 1973). The term Pulsus paradoxus (PP) was first used in 1873 by Adolf Kussmaul who described it as “a pulse slight and irregular disappearing during inspiration and returning upon expiration.” The current definition refers to an inspiratory fall in systolic blood pressure of more than 10 mmHg (Figure 3). To notice, the use of “paradoxal” should not be misconstrued, as the inspiratory drop in systolic pressure is physiological and not truly paradoxical in nature.

Currently, there is no consensus regarding the precise underlying physiological mechanism. Several factors may contribute to its occurrence in acute asthma. Among these factors, the main mechanism involves inspiratory decrease in ITP and high lung volume breathing. While the exact interplay of these factors remains subject to ongoing research.

Jardin et al. (1982) performed a hemodynamic study on a cohort of 9 patients with status asthmaticus, employing esophageal pressure monitoring, Swan-Ganz catheterization, echocardiography, and systemic arterial pressure. The results showed that esophageal pressure exhibited significant variations, ranging from negative levels during inspiration (−24.4 ± 6.5 cmH20) to positive level during expiration (7.6 ± 6.0 cm H20) due to hyperinflation-induced intrinsic PEEP (PEEPi). All patients exhibited tachycardia and increased cardiac index with low stroke volume. During inspiration, the decrease in radial artery pressure was greater in systolic than in diastolic pressure leading to a large and significant decrease in pulse pressure. Similar observations were made in pulmonary artery pressure (BRECHER and HUBAY, 1955). Both left and RV filling pressures increased during inspiration (i.e., pulmonary capillary wedge pressure and right atrial pressure) (BRECHER and HUBAY, 1955). The increase in right atrial pressure was three times greater than wedge pressure. Consequently, during inspiration, the filling pressure of RV exceeded that of the left ventricle, whereas during expiration, the opposite was observed (BRECHER and HUBAY, 1955). Echocardiography highlighted a significant decrease in end-systolic and end-diastolic left ventricular cross-sectional areas (respectively −24% and −32%) during inspiration compared to end expiratory values raising suspicion of a decreased stroke volume during inspiration (BRECHER and HUBAY, 1955). In contrast, RV end-systolic and end-diastolic diameters were increased (respectively 42% and 40%, *p* < 0.01) with septal flattening. In summary, during inspiration, left ventricular diameter decreased while RV diameter increased; with opposite changes occurring during expiration. These cyclic phenomena (negativation of pleural pressure, increase in vascular resistance linked to hyperinflation and variations in ventricular filling pressures during respiration) could contribute to the increase in hydrostatic pulmonary edema. The substantial decrease in diastolic pressure relative to Ppl suggests that passive transmission of ITP to left ventricle and indirectly along the arterial tree does not play a minor role in PP. A similar phenomenon was observed in the RV in seven asthmatic patients. An inspiratory RV enlargement coexisted with a reduction in two-dimensional echocardiographic stroke area and pulmonary artery pulse suggesting the impact of highly negative Ppl on the RV free wall (Jardin et al., 1987). Indeed, the negative pressure surrounding the RV during inspiration appears to be the main factor contributing to a reduction in the hydraulic force affecting RV ejection (Jardin et al., 1987).

Ultrasound findings indicate that PP during acute asthma may not be the only exaggeration of the inspiratory decrease in systolic arterial pressure as seen in healthy subjects. Notably, in both conditions, right atrial pressure does not exhibit elevation during expiration (Jardin et al., 1982). Venous return remains limited during deep inspiration by the vascular waterfall phenomenon. Simultaneously, the RV afterload increases leading to a decrease in right stroke area and pulmonary artery pulse pressure, as described above during hyperinflation with elevated PVR and increase in transmural pressure. These observations stand in contrast to the response observed in healthy subject (Jardin et al., 1987).

Contrary to previous findings, the decrease in RV stroke volume was not confirmed in a clinical study including asthmatic patients during a bronchial challenge mimicking acute asthmatic attack and producing PP (Blaustein et al., 1986). Ventricular volumes and ejection fractions were measured using radionuclide angiography during respiratory cycle. The RV stroke remained unchanged from baseline during inspiration. There was a notable increase in RV inspiratory end-diastolic volume with a decrease in left ventricular diastolic volume and LV stroke volume. These findings suggest that interdependence phenomenon between the LV and the RV plays a crucial role in the occurrence of PP. Supporting this notion, the experimental study by Gonzalez et al. (1991) in cardiac tamponade demonstrated that the two ventricles are 180° out of phase. Transposing to asthma, asthma where the stress on RV is also significant, adds weight to the argument for ventricular interdependence.

The influence of blood volume on cardio-pulmonary interactions during asthma is a relevant subject given the therapeutic implications. In this context, (Squara et al., 1990) conducted an interesting study involving 9 asthmatic patients presenting with acute symptomatology and paradoxical pulse (>20 mmHg). Pulsus paradoxus decreased during the inflation of military anti-shock trouser (MAST) and returned to baseline values after MAST deflation. The interpretation remains speculative. The decrease in PP was driven by an increase in inspiratory arterial pressure suspecting that the reduction of the venous return in asthma may have a greater influence on the occurrence of PP than the interplay of ventricular interdependence.

Given the divergent findings in literature, it seems relevant to defend the existence of multiple intricate mechanisms coexisting in severe acute asthma and contributing to the appearance of complex cardio-pulmonary interactions.

### Heart lung interactions during life-threatening asthma: management

In case of severe acute asthma, the therapies aim to improve respiratory mechanics by limiting or correcting airway narrowing to restore expiratory and inspiratory flows. Beta-2-agonists are recommended for their rapid action. Inhaled salbutamol is the bronchodilator of choice due to its effectiveness and tolerability, associated to ipratropium bromide in severe forms. The mechanism by which B2 agonists improve symptoms in asthma has been well described by Misouri *et al* (Misuri et al., 1997). Indeed, after histamine bronchial stimulation, the administration of fenoterol in 6 asthmatic subjects improved inspiratory muscle function during the acute phase, with a decrease in pleural pressure swings and dyspnea, however no effect on FEV1 was reported. This suggests that cardio-pulmonary interactions are improved mainly by reducing cyclic and profound variations in pleural pressure. Corticosteroid therapy is the treatment of choice to reduce the inflammatory component of asthma and to increase the sensitivity and the number of beta-2 adrenergic receptors.

Regarding the use of mechanical ventilation during acute asthma, some retrospective studies suggest potential benefits of noninvasive positive pressure ventilation, which could improve outcomes, particularly by avoiding the need for intubation (Alth et al., 2020). However, the role of this latter technique still needs to be clarified, and to date, is snot included in recent recommendations. Martin et al. investigated the effect of positive pressure ventilation on respiratory mechanics in 8 asthmatic patients after inducing bronchospasm with histamine. Positive pressure ventilation improved respiratory work, especially for the inspiratory muscles, with an increase in pleural pressure (Ppl) during inspiration from −32.3 (± 2.6 cm) H2O to −22.8 (± 2.3) cm H2O (*p* < 0.01) and reduced trans-diaphragmatic pressure variations (Martin et al., 1982). These results also suggest the potential benefits of limiting significant variations in pleural pressure to optimize the respiratory mechanics in this population. However, further studies are needed to confirm these findings. Indeed, positive pressure ventilation in asthmatic patients has been associated with an increased risk of barotrauma and pneumothorax. Inadvertent application of excessive positive end-expiratory pressure (PEEP) (higher than auto-PEEP) could contribute to dynamic hyperinflation and by consequence compromise hemodynamic.

Invasive mechanical ventilation is associated with a significant mortality in asthma (Brenner et al., 2009). Post-intubation hemodynamic instability is common, due to major lung hyperinflation, hypovolemia and sedation. Pulmonary hyperinflation might be important with the abolition of expiratory muscle activity which can lead to a decrease in expiratory flow rates. Therefore, orotracheal intubation should be carefully considered and limited to patients with life-threatening prognosis (respiratory arrest, bradypnea, coma, exhaustion, severe and refractory gazometric abnormalities). The objective of invasive ventilation is to ensure oxygenation while minimizing the risk of hyperinflation, which can be detrimental on the prognosis through cardio-pulmonary interactions and their consequences on the right ventricle (increased afterload, dilation, and tamponade via ventricular interdependence). Reducing tidal volume and lengthening expiratory time are accepted methods to achieve these objectives (Le Conte et al., 2019). In case of failure, circulatory assistance should be considered.

## Conclusion

Asthma, characterized by airway inflammation and narrowing, affects not only the lung mechanics but also exerts significant effect on cardiovascular system due to heart-lung interaction consequences. Healthcare providers should be aware of the intricate relationship between heart function and lungs to improve and optimize the management of acute asthma with a multidimensional approach that addresses concomitantly respiratory and hemodynamic aspects.

In high-risk patients, close monitoring of cardiac parameters (thermodilution devices or echocardiography) is crucial to adjust treatment strategies and to provide comprehensive care for patients suffering from near-fatal asthma. Future research should aim to elucidate and better understand the underlying complex mechanisms of heart lung interactions in a disease that still may kill young people in 2023.
